# Italian Olfactory Identification Test in Systemic Lupus Erythematosus: Association of Olfactory Impairment With Chronic Damage and Anti–β_2_‐Glycoprotein I Antibodies

**DOI:** 10.1002/acr2.90088

**Published:** 2026-06-21

**Authors:** Marta Di Berardino, Pasquale Nigro, Lorenza Bruno, Giacomo Cafaro, Anna Colangelo, Roberto Dal Pozzolo, Francesco Tromby, Roberto Gerli, Lucilla Parnetti, Elena Bartoloni, Nicola Tambasco, Carlo Perricone

**Affiliations:** ^1^ Section of Rheumatology, Department of Medicine and Surgery University of Perugia Perugia Italy; ^2^ Movement Disorders Center, Neurology Department Perugia General Hospital and University of Perugia Perugia Italy

## Abstract

**Objective:**

Olfactory dysfunction is a relatively frequent manifestation in systemic lupus erythematosus (SLE). The Italian Olfactory Identification Test (IOIT) may represent a suitable tool for detecting olfactory impairment in patients with SLE, due to its reliability and easiness of administration. This study aimed to evaluate the prevalence of smell impairment in patients with SLE and its correlation with clinical and serologic features.

**Methods:**

Consecutive patients with SLE and healthy controls were enrolled. Clinical history, disease indices, and main laboratory parameters were collected. Olfactory function was assessed using the IOIT, testing 33 cards with different microencapsulated odorants.

**Results:**

The cohort included 100 subjects: 50 patients with SLE (mean age ± SD: 51.3 ± 14.2; disease duration ± SD: 20.9 ± 13.2; female‐to‐male ratio: 5:1) and 50 age‐ and sex‐matched healthy controls. Hyposmia was observed in 30% of patients with SLE and 4% of healthy subjects. IOIT scores correlated significantly with cumulative organ damage measured by Systemic Lupus International Collaborating Clinics/American College of Rheumatology Damage Index (*P* = 0.017, r = 0.336). Hyposmia was associated with anti–β_2_‐glycoprotein I (anti‐β_2_GPI) antibodies (*P* = 0.029) and antidepressant therapy (*P* = 0.019). Cumulative damage was demonstrated to be an independent risk factor for hyposmia (odds ratio = 1.42, 95% confidence interval 1.02–1.98; *P* = 0.038).

**Conclusion:**

The IOIT appears to be a suitable tool for evaluating olfactory dysfunction in SLE. The significant association between hyposmia and cumulative damage suggests that olfactory impairment may represent a clinical marker of long‐term organ damage. The association between hyposmia and the use of antidepressant therapy confirms the close relationship between the sense of smell and mood regulation. Furthermore, the association with anti‐β_2_GPI reinforces the link between olfactory dysfunction and chronic damage in SLE.

## INTRODUCTION

The sense of smell is the most ancient sensory system in humans and plays a fundamental role in both species survival and social interaction.^1^, ^2^ A high prevalence of hyposmia has been reported in several diseases with distinct clinical and pathogenetic features, including multiple sclerosis, Parkinson disease (PD), hereditary angioedema, and systemic sclerosis.^3^, ^4^, ^5^, ^6^

Systemic lupus erythematosus (SLE) is a prototypical systemic autoimmune disease characterized by chronicity, clinical heterogeneity, and a broad spectrum of manifestations. Recent studies have suggested that olfactory dysfunction is relatively common in SLE: hyposmia has been reported in approximately 50% of patients,^3^, ^4^ whereas about 10% may experience anosmia.^3^ The prevalence appears to be higher in patients with neuropsychiatric SLE (NPSLE) compared with both healthy controls and patients without NPSLE^7^ and has been correlated with cognitive impairment and depression.^7^, ^8^ Interestingly, although olfactory dysfunction does not seem to be clinically evident in the early stages of SLE,^8^ functional magnetic resonance imaging studies have revealed reduced activation in olfactory‐related brain regions even at disease onset.^9^

The mechanisms underlying olfactory dysfunction in SLE remain incompletely understood. Potential contributors include anti–ribosomal P^4^, ^10^ and anti–double‐stranded DNA (anti‐dsDNA) antibodies,^9^ disease activity,^3^ systemic inflammation, and structural changes in olfactory‐processing brain regions such as the amygdala and hippocampus,^4^ as well as broader neuroimmunologic mechanisms.^11^

In this study, we investigated the prevalence of olfactory dysfunction in a monocentric cohort of patients with SLE and explored its associations with clinical and serologic features, with the aim of further characterizing this intriguing manifestation.

## MATERIALS AND METHODS

### Study population

A cohort of 50 patients with SLE fulfilling the 2019 EULAR/American College of Rheumatology (ACR) classification criteria was consecutively enrolled.^12^ Fifty age‐ and sex‐ matched healthy subjects were included as controls. Subjects with other medical conditions known to affect olfactory function, such as facial traumas or surgeries, active nasal or upper respiratory infections, head injuries, allergies, or SARS‐CoV‐2 infection in the previous six months, were excluded. Each patient underwent a thorough clinical evaluation and detailed medical history review, including age at diagnosis, disease duration, clinical manifestations according to ACR/EULAR clinical domains,^12^ smoking habits, diet, any dairy intolerance, anticoagulant, and antidepressant therapy. Current and previous therapies were recorded, including glucocorticoids (prednisone or equivalent), immunosuppressants (azathioprine, cyclophosphamide, cyclosporine A, voclosporin, mycophenolate mofetil, methotrexate, leflunomide, belimumab, anifrolumab, and rituximab), and hydroxychloroquine.

Disease activity, assessed by the Systemic Lupus Erythematosus Disease Activity Index 2000 (SLEDAI‐2K), and cumulative damage, assessed by the Systemic Lupus International Collaborating Clinics/ACR Damage Index (SDI), were recorded at enrollment. A SLEDAI‐2K score ≥4 was considered indicative of active disease.^13^

The following serologic variables were assessed at inclusion: antinuclear autoantibodies (ANAs) by indirect immunofluorescence, anti‐dsDNA and anti–extractable nuclear antigens (anti‐Ro/SSA, anti‐La/SSB, anti‐Sm, and anti‐RNP) by immunoblotting, antiphospholipid antibodies (IgG and IgM anticardiolipin antibody, IgG and IgM anti–β_2_‐glycoprotein I [anti‐β_2_GPI]) by enzyme‐linked immunosorbent assay, lupus anticoagulant by diluted Russel's viper venom time test, and complement factors C3 and C4 by nephelometry. Pathologic values were defined as follows: ANA ≥1:80; anti‐Ro/SSA, anti‐La/SSB, anti‐Sm, and anti‐RNP ≥15 U/mL; anti‐cardiolipin ≥40 IgM/IgG phospholipid units (MPL/GPL, respectively); anti‐β_2_GPI ≥40 MPL/GPL; and LAC ratio >1.20. Reduced complement levels were defined as serum C3 < 90 mg/dL and C4 < 15 mg/dL.

Patients were asked whether they had ever experienced any changes in their perception of taste or smell throughout the clinical course of the disease and specifically both during disease flares and just before performing the olfactory testing. Additionally, the questionnaire Beck's Depression Inventory I (BDI‐I) was administered to patients with SLE to assess mood status. The BDI‐I consists of a self‐administered questionnaire of 21 questions, each with four options describing increasing level of mood depression, scored from 0 to 3; the overall score is given by the sum of the points per single answer and ranges from 0 to 63, allowing patients to be stratified according to the severity of depression^14^, ^15^: mild (score 10–18), moderate (score 19–28), and severe (score 30–63). The study was approved by the local ethics committee (protocol number 11005/17/AV dated 20/07/2017, CEAS register number 3017/17), and all participants signed the informed consent form.

### Smell test: The IOIT

The Italian Olfactory Identification Test (IOIT) is based on the recognition of 33 different odorants typical of Italian culture. The patient is given 33 cards to smell (from number 1 to number 33), the suggested answers for each tester are read, and then the tester can be rubbed and sniffed. The patients have to choose one of the answers among the four suggested, crossing or circling it.^5^

The 33 IOIT odorants include clove, rose, lavender, banana, fir or pine tree, mushroom, talc, mint, coconut, strawberry sweet, apple, cheese, watermelon, fresh‐cut grass, violet flowers, sage, licorice, laundry soap, wood‐like smell, coffee, chocolate powder, oregano, basil, rosemary, garlic, lemon, peach, incense, orange, anise‐sambuca, pineapple juice, eucalyptus sweet, and unpleasant odor. Clove and mint are composed of single‐type volatile molecules (monomolecular odorant), whereas other odorants have a mixture of osmotically active molecules (multimolecular odorant).^5^ The substances used cover a broad spectrum of fragrances: fruity, floral, citrus, sweet, woody, fresh, spicy, and unpleasant.^5^ Hyposmia was determined based on the IOIT score and adjusted for age, using the following cutoffs: age range 30 to 49 years, score >4; 50 to 59 years, score >5; 60 to 69 years, score >6; and 70 to 79 years, score >7. The age standardization is necessary due to the progressive decline in smell sensitivity with age.^16^

### Statistical analysis

Data were presented as mean ± SD. The IOIT scores were calculated as total number of errors. Statistical analysis was conducted using the D'Agostino‐Pearson test to evaluate the distribution of the data, then the most suitable inference tests for such distributions were used. Descriptive and inferential statistics were used to summarize the data. Continuous variables were summarized by mean, SD, median, minimum, and maximum values. Categorical variables were summarized by counts and relative percentages.

First, a univariate analysis was performed for exploratory purposes, followed by a multivariate analysis to highlight the multiple relationships between the study variables. Then the Mann–Whitney test, chi‐square test, or Student's *t*‐test was used as appropriate. Statistical assessments of correlation were made by Pearson's R for parametric tests and Spearman's coefficient for nonparametric tests, and statistical assessments of association were made by odds ratio (OR). Observed patients were stratified according to the presence or absence of hyposmia. An initial univariate exploratory analysis was performed to identify potential associations, followed by a multivariable analysis to assess independent relationships among the study variables. Moreover, the positive correlation found between SDI and IOIT score was adjusted for age and for disease duration performing partial correlation and multivariable linear regression analysis. Two‐tailed *P* values ≤0.05 were considered significant.

Based on preliminary data showing an expected prevalence of olfactory impairment of approximately 30% in patients with SLE and 2% in healthy controls, with a two‐sided α of 0.05 and 80% power, at least 21 subjects per group were required. Therefore, the inclusion of 50 patients with SLE and 50 controls was considered adequate for detecting a between‐group difference in IOIT impairment prevalence. The number of patients was limited to perform multivariable analysis that was intentionally restricted to two clinically relevant covariates in order to minimize overfitting. Logistic regression analysis was performed using a parsimonious modeling strategy, and penalized regression methods were considered to account for the relatively small sample size, whereas correlation analyses using the continuous IOIT score were performed to preserve statistical information.

Data were analyzed using GraphPad Prism version 8 software (GraphPad Software, Inc) and IBM SPSS Statistics for Windows, version 20.0 (IBM Corp).

## RESULTS

A total of 100 subjects was enrolled in the study. The cohort of patients with SLE included 50 individuals with a mean age ± SD of 51.3 ± 14.2, mean disease duration ± SD of 20.9 ± 13.21, and female‐to‐male ratio of 5:1. The cohort of healthy controls included 50 individuals with a mean age ± SD of 54.26 ± 11.6 and female‐to‐male ratio of 5:1. Laboratory findings, clinical features, and treatments at baseline are summarized in Table 1. According to the BDI questionnaire, 18 patients with SLE had mild depression, 5 had moderate depression, and none had severe depression (BDI > 30). Nineteen patients (38%) had active disease (SLEDAI ≥ 4), whereas 41 patients (82%) showed cumulative organ damage (SDI ≥ 1).

**Table 1:** Descriptive analysis of the SLE cohort (n = 50)*

	Value
Sex, n (%)	
Male	8 (16)
Female	42 (84)
Age, mean ± SD, y	51.3 ± 14.2
Age at diagnosis, mean ± SD, y	30.5 ± 13.7
Disease duration, mean ± SD, y	20.9 ± 13.2
Xerostomia, n (%)	19 (38)
Xerophthalmia, n (%)	17 (34)
Arthritis, n (%)	12 (24)
Photosensitivity, n (%)	5 (10)
Malar rash, n (%)	3 (6)
Aphthous or ulcers, n (%)	1 (2)
Pericarditis, n (%)	0 (0)
Previous pericarditis, n (%)	14 (28)
Pleuritis, n (%)	0 (0)
Previous pleuritis, n (%)	11 (22)
Anemia, n (%)	7 (14)
History of anemia, n (%)	30 (60)
Leukopenia, n (%)	3 (6)
History of leukopenia, n (%)	23 (46)
Thrombocytopenia, n (%)	1 (2)
History of thrombocytopenia, n (%)	17 (34)
NPSLE ever, n (%)	14 (28)
Kidney involvement, n (%)	26 (52)
COVID‐19 ever, n (%)	26 (52)
Former smokers, n (%)	11 (22)
Active smokers, n (%)	9 (18)
Never smokers, n (%)	30 (60)
Diet (vegetarian, vegan, keto), n (%)	1 (2)
Smell changes, n (%)	11 (22)
Taste changes, n (%)	10 (20)
ANA, n (%)	50 (100)
Anti‐dsDNA, n (%)	32 (64)
Anti‐ENA, n (%)	34 (68)
Anti‐Sm, n (%)	16 (32)
Anti‐RNP, n (%)	13 (26)
Anti‐SSA, n (%)	19 (38)
Anti‐SSB, n (%)	6 (12)
Anticardiolipin, n (%)	9 (18)
Anti‐β_2_GPI, n (%)	8 (16)
LAC, n (%)	9 (18)
Low C3, n (%)	23 (46)
Low C4, n (%)	24 (48)
Antidepressant therapy, n (%)	11 (22)
ASA, n (%)	12 (24)
Anticoagulants, n (%)	4 (8)
Prednisone, n (%)	15 (30)
HCQ, n (%)	39 (78)
MMF, n (%)	14 (28)
MTX, n (%)	5 (10)
AZA, n (%)	1 (2)
VOCLO, n (%)	1 (2)
Biologic therapy, n (%)	7 (14)
RTX, n (%)	2 (4)
BLM, n (%)	4 (8)
ANF, n (%)	1 (2)

* ANA, antinuclear antibody; ANF, anifrolumab; anti‐β_2_GPI, anti–β_2_ glycoprotein I; anti‐dsDNA, anti–double‐stranded DNA; anti‐ENA, anti–extractable nuclear antigen; ASA, acetylsalicylic acid; AZA, azathioprine; BLM, belimumab; HCQ, hydroxychloroquine; LAC, lupus anticoagulant; MMF, mycophenolate mofetil; MTX, methotrexate; NPSLE, neuropsychiatric systemic lupus erythematosus; RTX, rituximab; SLE, systemic lupus erythematosus; VOCLO, voclosporin.

**Figure 1:**
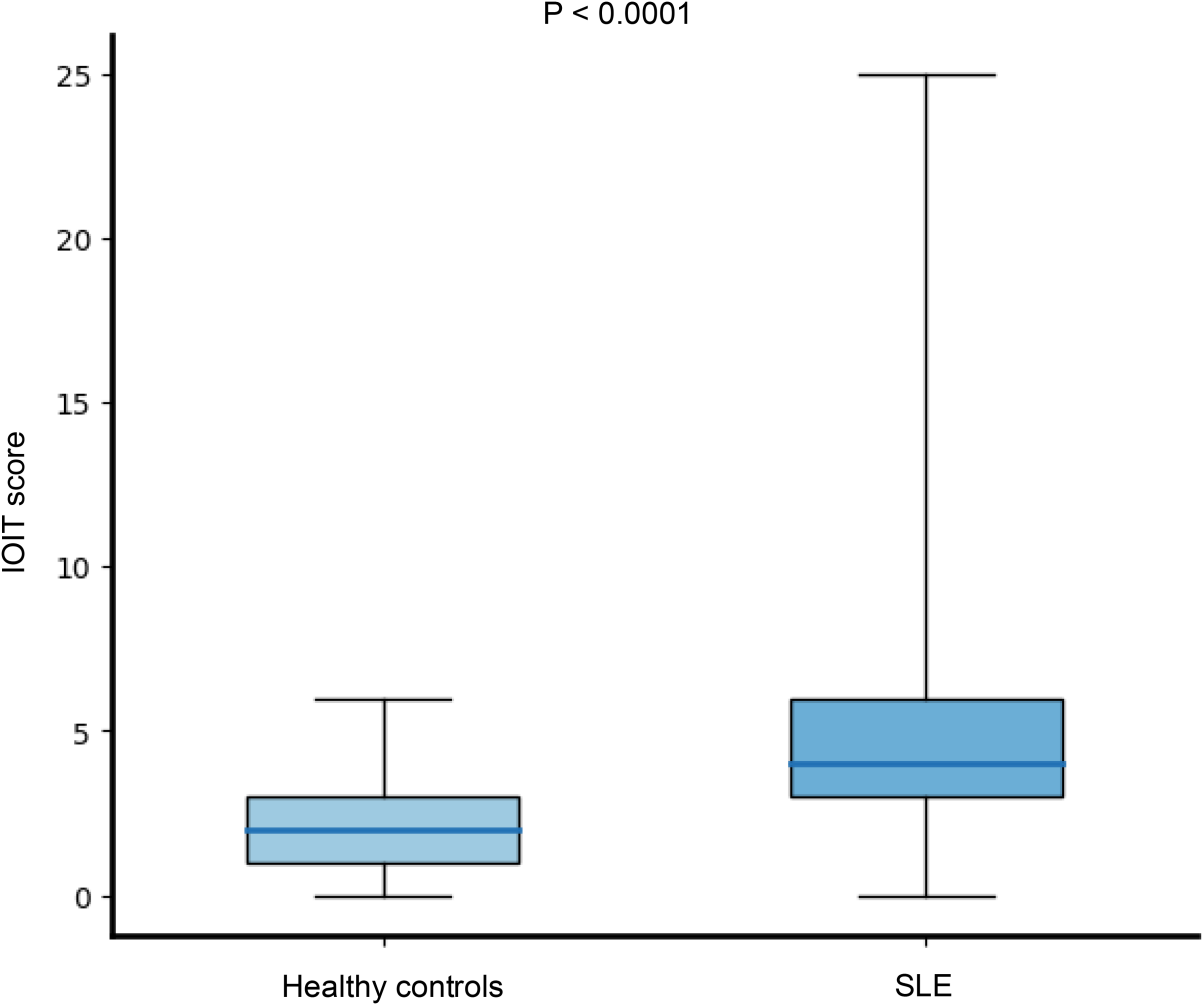
IOIT scores in patients with SLE and healthy controls (*P* < 0.0001). IOIT, Italian Olfactory Identification Test; SLE, systemic lupus erythematosus.

**Figure 2:**
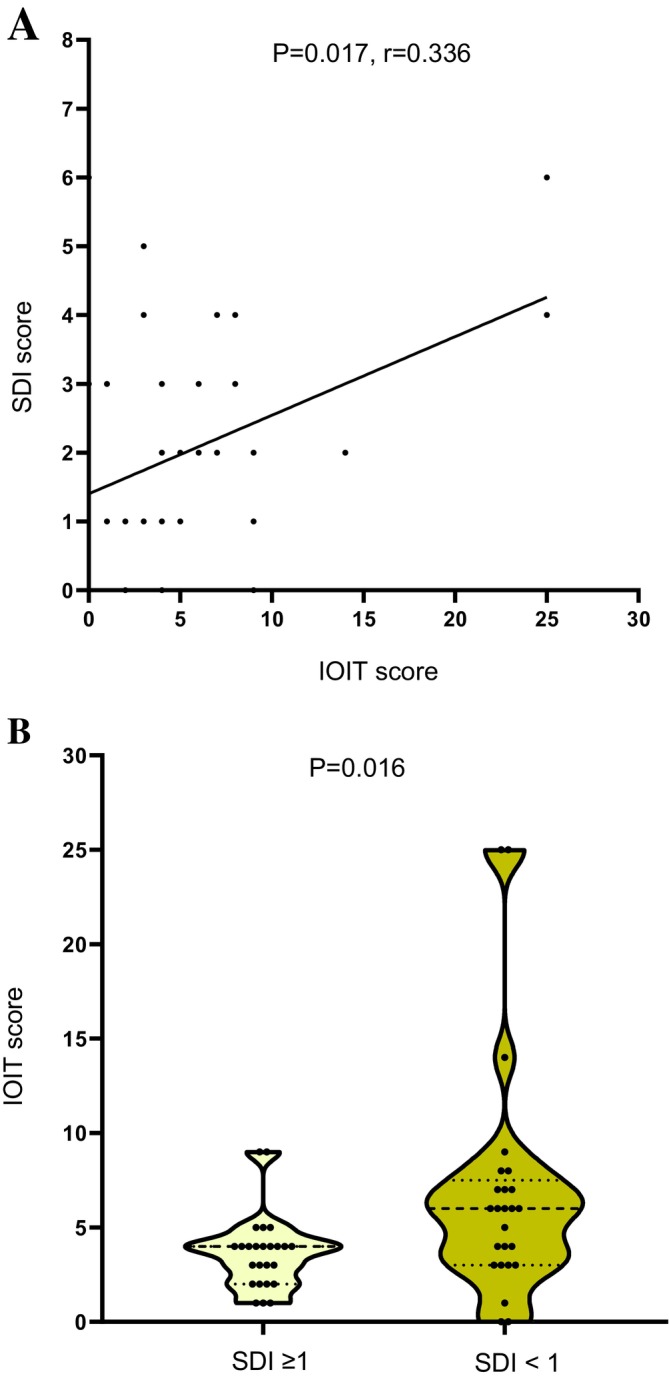
IOIT–SDI correlation. (A) IOIT score significantly correlated with cumulative organ damage assessed by SDI (*P* = 0.017, r = 0.336). (B) Patients with SDI >1 had a significantly higher mean number of errors in IOIT compared to those with SDI ≤1 (*P* = 0.016). IOIT, Italian Olfactory Identification Test; SDI, Systemic Lupus International Collaborating Clinics/American College of Rheumatology Damage Index.

**Figure 3:**
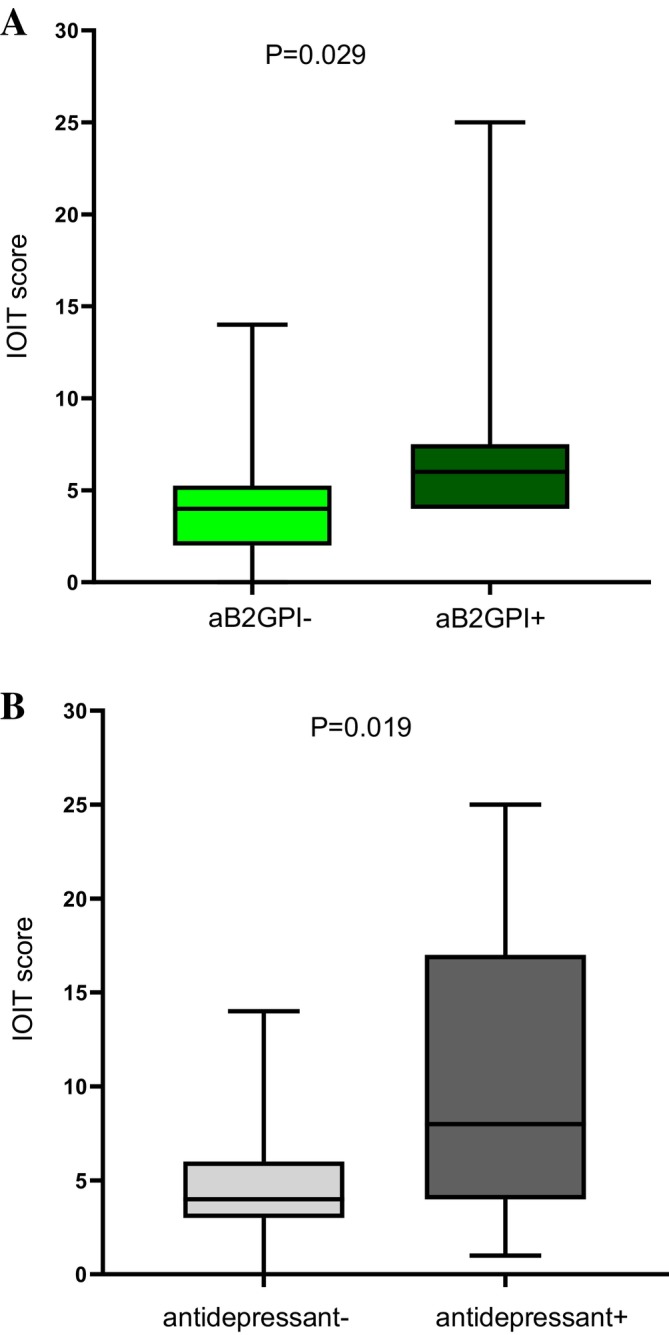
Association between olfactory impairment and clinical variables. (A) aB2GPI antibody positivity (*P* = 0.029). (B) Antidepressant therapy (*P* = 0.019). aB2GPI, anti–β_2_ glycoprotein I; IOIT, Italian Olfactory Identification Test.

The prevalence of hyposmia, defined as a higher number of errors at IOIT than expected for their age group, was significantly higher in patients with SLE compared to healthy controls (15/50 SLE vs 2/50 controls, *P* < 0.001). Overall, patients with SLE had significantly higher IOIT scores (indicating reduced sense of smell) compared to healthy subjects (*P* < 0.0001; Figure 1). When stratified by age (<40, 40–59, and >60 years), patients with SLE showed significantly higher IOIT scores than healthy controls across all age ranges (<40 years: *P* = 0.009; 40–59 years: *P* = 0.006; and >60 years: *P* = 0.001), as shown in Table 2. In the SLE cohort, a significant positive correlation was found between IOIT scores and SDI values (*P* = 0.017, r = 0.336; Figure 2A). This positive correlation remained significant after adjustment for age (partial r = 0.283, *P* = 0.047; Supplementary Figure 1). In multivariable linear regression analysis including age and SDI, cumulative organ damage remained independently associated with impaired odor identification (β = 0.82 per point, *P* = 0.049), whereas age was no longer significant. Moreover, the association between IOIT and SDI persisted after adjustment for disease duration (partial r = 0.281, *P* = 0.048; Supplementary Figure 2). In multivariable analysis including both SDI and disease duration, SDI retained an independent effect (β = 0.81 per point, *P* = 0.050), whereas disease duration did not remain significant. Furthermore, patients with SDI >1 had a significantly higher mean number of IOIT errors (*P* = 0.016; Figure 2B) and higher prevalence of hyposmia (χ^2^ = 6.10, *P* = 0.014; Supplementary Table 1) compared with those with SDI ≤1. Patients were then stratified according to IOIT scores: demographic and clinical data of normosmic versus hyposmic patients are presented in Supplementary Table 2. Antidepressant therapy and anti‐β_2_GPI antibodies showed an association with olfactory dysfunction (*P* = 0.019 and *P* = 0.029, respectively), as illustrated in Figure 3A and B. The use of antidepressant therapy was significantly associated with both BDI scores (*P* = 0.006) and the severity of depression (*P* = 0.013).

**Table 2:** IOIT results in healthy controls and patients with SLE stratified according to the age range*

Age range, y	HC (n = 50)				SLE (n = 50)				*P* value
	n	Mean IOIT	SD	Hyposmic	n	Mean IOIT	SD	Hyposmic	
<40	7	1.14	0.9	0	12	3.67	2.27	3	0.009
40–59	22	2.23	1.51	2	20	5.45	5.6	8	0.006
>60	21	2.57	1.83	0	18	6.06	5.21	4	0.001

* Patients with SLE showed significantly higher IOIT scores than healthy controls across all age ranges. HC, healthy control; IOIT, Italian Olfactory Identification Test; SLE, systemic lupus erythematosus.

In multivariable logistic regression analysis, higher cumulative organ damage, as assessed by the SDI, was independently associated with impaired odor identification (OR 1.42 per point, 95% confidence interval [CI] 1.02–1.98; *P* = 0.038). Age, disease duration, complement consumption (low C3 or C4), and anti‐β_2_GPI positivity were not independently associated with olfactory impairment (Table 3).

**Table 3:** Multivariable logistic regression analysis*

Variable	OR	95% CI	*P* value
Age (per year)	1.03	0.97–1.10	0.29
Disease duration (per year)	1.01	0.97–1.05	0.63
Low C3	2.36	0.56–9.93	0.24
Low C4	1.92	0.39–9.41	0.42
SDI	**1.42**	**1.02–1.98**	**0.038**
Anti‐β_2_GPI positivity	2.87	0.74–11.13	0.13

* Higher cumulative organ damage, as assessed by the SDI, was independently associated with impaired odor identification (OR 1.42 per point, 95% CI 1.02–1.98; *P=*0.038). Age, disease duration, complement consumption (low C3 or C4), and anti‐β_2_GPI positivity were not independently associated with olfactory impairment. Bold values indicate significant association. Anti‐β_2_GPI, anti–β_2_ glycoprotein I; CI, confidence interval; OR, odds ratio; SDI, Systemic Lupus International Collaborating Clinics/American College of Rheumatology Damage Index.

## DISCUSSION

In this study, the IOIT has been confirmed as a reliable and easy‐to‐administer tool to assess olfactory function in both healthy individuals and patients with SLE. These findings are in line with what reported by Maremmani et al in their previous validation study of the IOIT in a PD population.^5^ Compared with other olfactory testing methods,^3^, ^4^, ^7^, ^8^, ^9^ the IOIT overcomes several methodologic limitations. The testing cards are identical in shape and color (white square cards), minimizing potential visual interference during response selection. In addition, answer options are presented directly in front of the participant, reducing reliance on working memory, which could otherwise influence odor identification performance.

Moreover, consistent with previous reports, we confirmed that a considerable proportion of patients with SLE have hyposmia as compared to healthy individuals.^2^, ^3^, ^4^, ^6^, ^8^ The prevalence observed in our cohort (30%) was slightly lower than that reported in the literature, which ranges from 37.1% in the study by Xu et al, which included both hyposmic and anosmic patients)^8^ to 46% in the cohort described by Shoenfeld et al,^3^ and up to 54.5% in the study by Bombini et al.^4^ In this setting, the different methods used to quantify olfactory dysfunction across these studies may partly limit the reliability of direct comparisons. In our study, we used the IOIT considering the lack of validated olfactometric tools for SLE, its specificity for typical Italian fragrances, and its recent validation in PD, another condition involving the central nervous system.^5^ Moreover, the use of an olfactory test specifically tailored to the cultural background in which patients were born or have predominantly lived may help improve test specificity, potentially reducing errors related to unfamiliarity with the odorant evaluated.

Several studies have explored the relationship between hyposmia and neurologic involvement in SLE, consistently showing a link between neuropsychopathologic manifestations and olfactory impairment.^7^, ^8^, ^9^ In these studies, different approaches have been employed, including the Connecticut Chemosensory Clinical Research Center method in the study by Chen et al,^9^ computerized testing for odor threshold, identification, and memory in the study by Xu et al,^8^ and the Brief Smell Identification Test in the study by Cavaco et al.^7^ Despite methodologic variability, olfactory dysfunction emerges as a prevalent and consistent finding in SLE, warranting further investigation into its underlying pathogenesis.

To the best of our knowledge, our study provided the first demonstration that olfactory dysfunction is associated with cumulative disease‐related organ damage. Patients with any damage (SDI > 1) showed a higher risk of hyposmia, suggesting that olfactory impairment in SLE may reflect the chronic damage accrued over time. Given the known increase in olfactory dysfunction with advancing age,^5^ we explored whether the observed association between IOIT scores and cumulative organ damage could be confounded by age or by disease duration. After adjustment both for age and disease duration using partial correlation and multivariable linear regression analysis, the association between SDI and olfactory impairment remained statistically significant. These findings indicate that cumulative organ damage is independently associated with impaired odor identification. Although we could not identify which specific SDI domains were most closely linked to hyposmia, steroid treatment can likely be excluded because both current and past use were assessed without association. Thus, hyposmia does not seem to represent an early manifestation of SLE but rather an easily measurable indicator of accumulated disease burden.

An additional explanation may lie in the observed association between hyposmia and anti‐β_2_GPI antibody positivity. These antibodies have been shown to increase SDI scores in SLE^17^, ^18^ and can potentially damage olfactory pathways through thrombotic mechanisms.^19^, ^20^ Although Bombini et al did not find any association between antiphospholipid antibody and olfactory dysfunction,^4^ the specific antibody profile and frequency in that study were not reported, which may partly explain the discrepancy.

We could not confirm the correlation between hyposmia and disease activity assessed by SLEDAI, as reported by Shoenfeld et al.^3^ Although the SLEDAI score was higher in hyposmic patients, the limited number of active cases likely reduced statistical power. Similarly, no association between hyposmia and NPSLE was observed, probably due to the low prevalence of neurologic involvement in our cohort.

Interestingly, we also found that antidepressant therapy is associated with hyposmia. Although no direct association with depression was identified in our population, likely due to the low number of patients with mild‐to‐moderate depression, the link between olfactory dysfunction and depression is well established.^7^ Anatomically, the neural circuits regulating mood and emotion (Papez circuit and limbic system) overlap with those involved in olfactory processing, particularly the orbitofrontal cortex.^7^, ^21^, ^22^ This neuroanatomical interplay may underlie the bidirectional relationship between mood disorders and olfactory impairment and opens the possibility of exploring aromatherapy as a nonpharmacologic therapeutic approach for depression.^23^ On the other hand, a direct pharmacologic effect of antidepressants appears unlikely because patients were treated with different drug classes, including selective serotonin reuptake inhibitors and serotonin and norepinephrine reuptake inhibitors. Further supporting this observation, antidepressant therapy was significantly associated with BDI scores and severity grading of depression in our cohort.

Finally, no association was found between olfactory impairment and smoking history, consistent with Maremmani et al.^5^ We hypothesize that the IOIT's ability to test a wide range of fragrances minimizes the confounding effect of smoking status, making it a suitable tool for studies including smokers.

Among the strengths of our study is the long disease duration of our cohort, which allowed us to capture the long‐term impact of SLE and robustly assess the link between olfactory dysfunction and cumulative organ damage. The present study, however, has some limitations that should be acknowledged. First, the relatively limited sample size and the low number of olfactory impairment events restricted the complexity of multivariable analyses, which were therefore intentionally limited to a parsimonious two‐covariate model to reduce the risk of overfitting. Consequently, the present findings should be considered exploratory, and larger prospective studies are warranted to externally validate these observations. Finally, it should be noted that the IOIT is not able to distinguish anosmia or grade severity, restricting our analysis to the presence or absence of olfactory dysfunction and it does not assess olfactory threshold. Nevertheless, unlike other olfactory tests such as the Sniffin’ Sticks test used in the studies by Bombini et al and Shoenfeld et al,^3^, ^4^ the IOIT includes odorants validated for the Italian population, minimizing the risk of odor misidentification related to cultural unfamiliarity.

In conclusion, olfactory dysfunction is relatively frequent in SLE, and the IOIT appears to be a suitable tool to assess olfactory dysfunction in patients with SLE, given its easiness of administration, noninvasive nature, good tolerability, time efficiency, and suitability for use in heterogeneous populations. Hyposmia in patients with SLE highlights the intriguing interplay between olfaction and autoimmunity. The observed association between olfactory dysfunction and cumulative organ damage suggests that hyposmia may serve as a marker of chronic disease‐related damage rather than an early manifestation and it may reflect long‐term disease burden in SLE. Further studies with neuroimaging data are warranted to clarify the underlying mechanisms. Such research may deepen our understanding of SLE pathogenesis and ultimately improve disease monitoring and patient management.

## AUTHOR CONTRIBUTIONS

All authors contributed to at least one of the following manuscript preparation roles: conceptualization AND/OR methodology, software, investigation, formal analysis, data curation, visualization, and validation AND drafting or reviewing/editing the final draft. As corresponding author, Prof Bartoloni confirms that all authors have provided the final approval of the version to be published and takes responsibility for the affirmations regarding article submission (eg, not under consideration by another journal), the integrity of the data presented, and the statements regarding compliance with institutional review board/Declaration of Helsinki requirements.

## Supporting information


**Disclosure Form**:

**Supplementary Figure 1** Partial regression plot showing the independent association between SDI and IOIT after adjustment for age (partial r=0.283, P=0.047)
**Supplementary Figure 2**. Partial regression plot showing the independent association between SDI and IOIT after adjustment for disease duration (partial r=0.281, P=0.048)

**Supplementary Table 1** Stratification according to SDI >1 confirmed a significantly higher prevalence of hyposmia among patients with greater cumulative organ damage (χ^2^ = 6.10, P=0.014)
**Supplementary Table 2**. Demographic and clinical outcomes in normosmic vs hyposmic patients; SLE, Systemic Lupus Erythematosus; SD, standard deviation; SLEDAI, Systemic Lupus Erythematosus Disease Activity Index; SDI, SLICC Damage Index; BDI, Beck's Depression Inventory; IOIT, Italian Olfactory Identification Test.
