# Supplementary material for: Italian Olfactory Identification Test in Systemic Lupus Erythematosus: Association of Olfactory Impairment With Chronic Damage and Anti–β2‐Glycoprotein I Antibodies
Source: ACR Open Rheumatol. 2026 Jun 21;8(6):e90088. doi: 10.1002/acr2.90088 (PMC13283763; doi:10.1002/acr2.90088)
Supplement: Supplementary file 2 — Supplementary Figure 1 Partial regression plot showing the independent association between SDI and IOIT after adjustment for age (partial r=0.283, P=0.047) Supplementary Figure 2. Partial regression plot showing the independent association between SDI and IOIT after adjustment for disease duration (partial r=0.281, P=0.048) [file ACR2-8-e90088-s001.pdf]

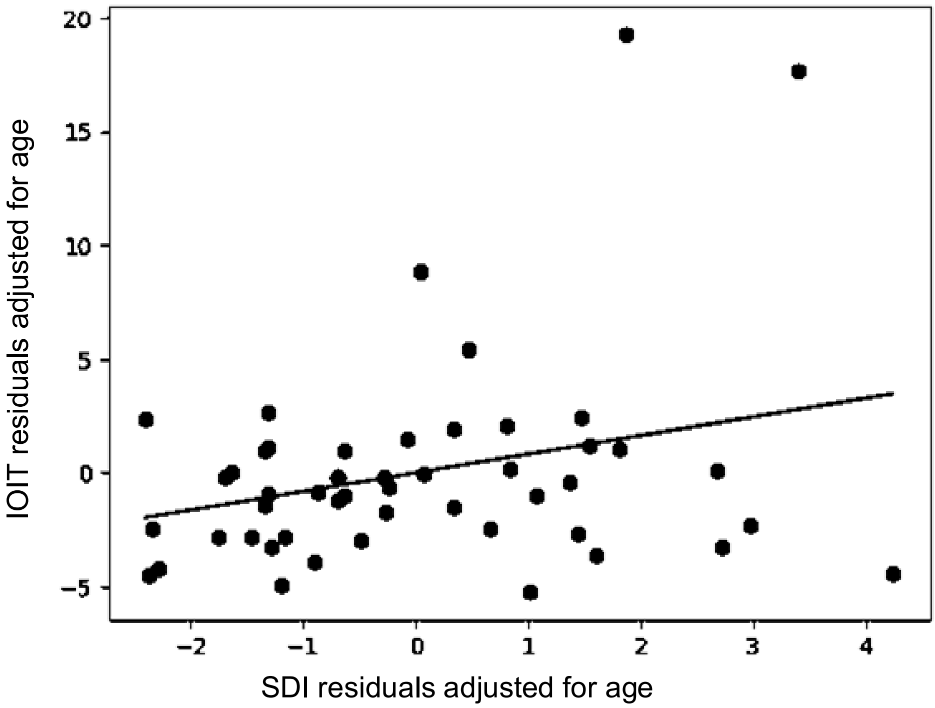

Supplementary Figure 1. Partial regression plot showing the independent association between SDI and IOIT after adjustment for age (partial r=0.283, P=0.047)

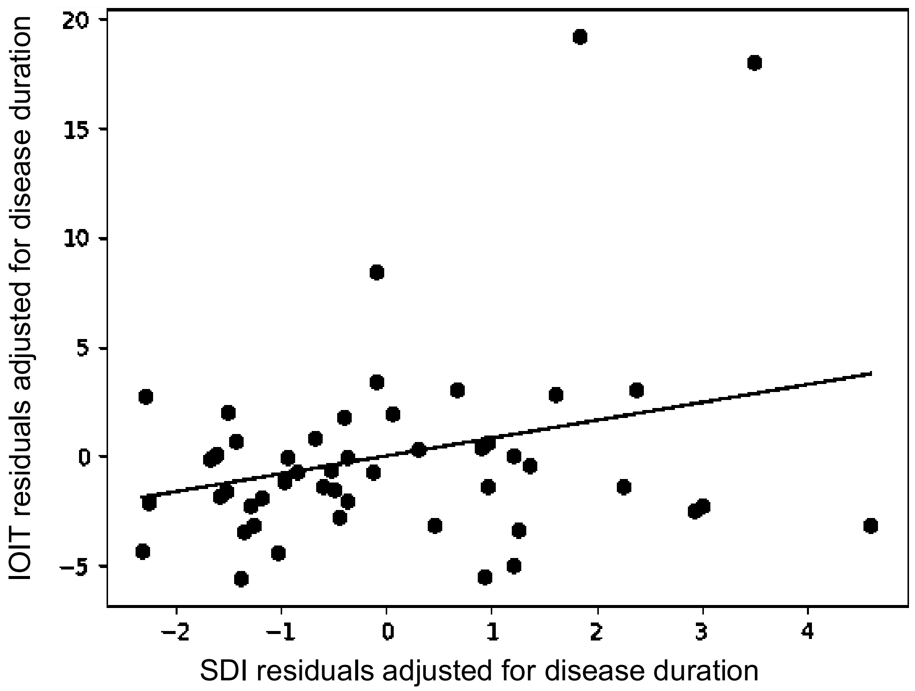

Supplementary Figure 2. Partial regression plot showing the independent association between SDI and IOIT after adjustment for disease duration (partial r=0.281, P=0.048)
