# Supplementary material for: Italian Olfactory Identification Test in Systemic Lupus Erythematosus: Association of Olfactory Impairment With Chronic Damage and Anti–β2‐Glycoprotein I Antibodies
Source: ACR Open Rheumatol. 2026 Jun 21;8(6):e90088. doi: 10.1002/acr2.90088 (PMC13283763; doi:10.1002/acr2.90088)
Supplement: Supplementary file 3 — Supplementary Table 1 Stratification according to SDI >1 confirmed a significantly higher prevalence of hyposmia among patients with greater cumulative organ damage (χ2 = 6.10, P=0.014) Supplementary Table 2. Demographic and clinical outcomes in normosmic vs hyposmic patients; SLE, Systemic Lupus Erythematosus; SD, standard deviation; SLEDAI, Systemic Lupus Erythematosus Disease Activity Index; SDI, SLICC Damage Index; BDI, Beck's Depression Inventory; IOIT, Italian Olfactory Identification Test. [file ACR2-8-e90088-s002.docx]

|  | Normosmia | Hyposmia |
| --- | --- | --- |
| SDI ≤1 | 22 | 3 |
| SDI>1 | 13 | 12 |

**Supplementary Table 1.** Stratification according to SDI >1 confirmed a significantly higher prevalence of hyposmia among patients with greater cumulative organ damage (χ² = 6.10, P=0.014)

| **SLE cohort** | **NORMOSMIC (n=35)** | | **HYPOSMIC (n=15)** | |  |
| --- | --- | --- | --- | --- | --- |
|  | Mean | SD | Mean | SD | P value |
| Age (years) | 50.51 | 15.23 | 53.13 | 11.64 | NS (0.51) |
| Disease duration (years) | 18.83 | 12.51 | 25.80 | 13.94 | NS (0.10) |
| SLEDAI | 2.66 | 3.37 | 4.13 | 5.44 | NS (0.34) |
| SDI | 1.74 | 1.67 | 2.60 | 1.5 | NS (0.08) |
| IOIT | 3.23 | 1.63 | 9.93 | 6.51 | <0.0001 |
| BDI | 10.34 | 7.46 | 9.79 | 6.86 | NS (0.8) |

**Supplementary Table 2.** Demographic and clinical outcomes in normosmic vs hyposmic patients; SLE, Systemic Lupus Erythematosus; SD, standard deviation; SLEDAI, Systemic Lupus Erythematosus Disease Activity Index; SDI, SLICC Damage Index; BDI, Beck's Depression Inventory; IOIT, Italian Olfactory Identification Test.
